# Ischemic Brain Injury: Involvement of Lipids in the Pathophysiology of Stroke and Therapeutic Strategies

**DOI:** 10.3390/antiox13060634

**Published:** 2024-05-23

**Authors:** Nathalie Bernoud-Hubac, Amanda Lo Van, Adina-Nicoleta Lazar, Michel Lagarde

**Affiliations:** Univ Lyon, INSA Lyon, CNRS, LAMCOS, UMR5259, 69621 Villeurbanne, France; amanda.lo-van@insa-lyon.fr (A.L.V.); adina-nicoleta.lazar@insa-lyon.fr (A.-N.L.); michel.lagarde@insa-lyon.fr (M.L.)

**Keywords:** lipids, brain, ischemic stroke, oxidative stress, therapeutic strategies

## Abstract

Stroke is a devastating neurological disorder that is characterized by the sudden disruption of blood flow to the brain. Lipids are essential components of brain structure and function and play pivotal roles in stroke pathophysiology. Dysregulation of lipid signaling pathways modulates key cellular processes such as apoptosis, inflammation, and oxidative stress, exacerbating ischemic brain injury. In the present review, we summarize the roles of lipids in stroke pathology in different models (cell cultures, animal, and human studies). Additionally, the potential of lipids, especially polyunsaturated fatty acids, to promote neuroprotection and their use as biomarkers in stroke are discussed.

## 1. Introduction

Hypoxic–ischemic brain damage (HIBD) results in partial or complete hypoxia of brain tissue due to decreased or suspended cerebral blood flow. Among HIBD, ischemic stroke is a leading cause of mortality and disability worldwide [1,2]. Strokes mainly affect the brain. Still, in less than 1.5% of cases, the occlusion of the blood flow to the spine may also occur, leading to spinal strokes. Cerebral stroke can be classified into hemorrhagic stroke and ischemic stroke, the latter accounting for 80 to 85% of cases. Lipids play critical roles in maintaining the structural integrity, energy metabolism, and signaling pathways in the brain. Dysregulation of lipid metabolism has been implicated in the pathogenesis of stroke [3,4,5,6]. Ischemia–reperfusion injury leads to the excessive production of reactive oxygen species (ROS), resulting in lipid, protein, and DNA oxidation. Lipid-derived oxidative stress contributes to neuronal injury, blood–brain barrier disruption, and neuroinflammation in stroke. Targeting lipid-mediated mechanisms represents a promising therapeutic approach for stroke prevention and treatment. Understanding the relationship between ischemic stroke and lipids is essential for elucidating potential preventive and therapeutic strategies.

This review aims to describe the mechanisms involved in the pathophysiology of hypoxic–ischemic brain injury, especially cerebral stroke, with a focus on the importance of reactive oxygen species production and lipid peroxidation that can lead to the clinical condition of hypoxic–ischemic. Highlighting the roles of lipids in these pathologies could help to define lipid-based strategies for the prevention of stroke or treatment.

## 2. Stroke

Stroke, also known as cerebrovascular accident (CVA), is the second leading cause of death and disability worldwide [1,2] with 16 million incidents, an annual mortality rate of about 6.6 million, and 50% of survivors being chronically disabled [7,8,9]. Both in Europe and the USA, stroke has been the leading neurological disease in terms of disability-adjusted life years (DALYs) [10,11,12]. Alarming studies showed a less favorable trend in the incidence of stroke in younger vs. older individuals [13,14,15,16]. Globally, between 1990 and 2013, there was a significant increase in the absolute number and prevalence rate of stroke for younger adults [17]. Studies predict that stroke mortality worldwide will increase by 50%, from 6.6 million in 2020 to 9.7 million in 2050, and that DALYs will rise over the same period, from 144.8 million in 2020 to 189.3 million in 2050 [1]. Age is the most important demographic risk factor of stroke, and the lifetime risk of stroke has increased with aging [18]. Ethnic differences also exist in the prevalence of stroke risk factors [19,20]. The major risk factors for stroke are previous stroke or transient ischemic attack, hypertension (being the most significant risk factor for stroke), diabetes mellitus, obesity, hyperlipidemia, smoking, and drug abuse [21,22,23,24].

A stroke occurs when the blood supply to some part of the brain is interrupted or reduced, depriving brain tissue of oxygen and nutrients. Strokes can be classified into two major categories: hemorrhagic stroke and ischemic stroke. Both types of strokes impair the correct functioning of the brain. Hemorrhagic strokes occur when a weakened blood vessel ruptures and bleeds into the surrounding brain tissue [25,26,27]. Hemorrhagic stroke may be further subdivided into two main types including intracerebral hemorrhage (ICH), when blood spills into the surrounding tissue following the rupture of a blood vessel, causing damage to nearby cells, and subarachnoid hemorrhage (SAH), when bleeding occurs into the space between the brain and the surrounding membrane (the arachnoid membrane). Hemorrhagic stroke is associated with severe morbidity and high mortality [28]. The symptoms of a hemorrhagic stroke may include severe increases in blood pressure, sudden severe headache, vomiting, agitation, and mydriatic pupil [29]. The treatment for hemorrhagic stroke may involve surgery to repair blood vessels, medications to reduce blood pressure and prevent further bleeding, and supportive care to manage symptoms and prevent complications [30].

Ischemic stroke, also known as cerebral ischemia, generally accounts for about 80% to 85% of all strokes and is the third leading cause of death worldwide, with nearly 15 million people affected yearly [29,31]. Ischemic strokes occur when a blood clot blocks or narrows an artery leading to the brain, reducing blood flow to a part of the brain. There are two main types of ischemic strokes including thrombotic stroke, when a blood clot (thrombus) develops within a blood vessel supplying blood to the brain, and embolic stroke, when a blood clot or other debris forms elsewhere in the body (often in the heart, cardioembolic stroke, or large arteries) and travels through the bloodstream to the brain. Ischemic stroke may have various clinical manifestations including vomiting, paresis, paralysis, ataxia, acute headache, and agitation [29].

The main therapeutic approaches in patients with acute cerebral ischemia are reperfusion treatments, either via mechanical thrombectomy or more commonly via intravenous thrombolysis. Intravenous thrombolysis or intravenous alteplase, with tissue-type plasminogen activator (tPA), which is aimed at restoring the cerebral blood flow, is restricted to few patients because of a narrow therapeutic window of 4.5 h and a high hemorrhagic risk [32,33]. Understanding the mechanisms involved in the pathophysiology of stroke is crucial for developing effective interventions to prevent and treat ischemic stroke. The interruption of blood flow deprives the affected brain tissue of oxygen and nutrients, leading to cellular dysfunction and death. A cascade of pathophysiological and interconnected changes occurs, including pathological permeability of the blood–brain barrier (BBB), neuroinflammation, neuronal apoptosis, autophagy, excitotoxicity, and ionic imbalance, as well as oxidative stress. 

## 3. Oxidative Stress and Ischemic Stroke

During an ischemic stroke, the lack of oxygen and nutrients supplied to the brain cells can lead to the production of free radicals, resulting in oxidative stress. Free radicals are highly reactive molecules that contain oxygen and can cause damage to cells, proteins, lipids, and DNA. This oxidative stress can exacerbate the damage caused by the stroke and contribute to further injury to brain tissue. Oxidative stress, characterized by the overproduction of reactive oxygen species (ROS) and impaired antioxidant defenses, represents a hallmark feature of ischemic stroke pathology [34,35,36]. Reactive nitrogen species (RNS) are also predominantly involved in the pathogenesis of stroke. The brain is particularly vulnerable to ROS and RNS compared with other organs because of high oxygen consumption, low neuronal antioxidant activity, high levels of peroxidizable lipids, and high concentrations of iron [37,38]. Ischemia–reperfusion injury triggers the excessive production of ROS, including superoxide anions (O_2_^−^), hydroxyl radicals (OH^−^), and hydrogen peroxide (H_2_O_2_), primarily through the activation of nicotinamide adenine dinucleotide phosphate (NADPH) oxidase, xanthine oxidase, and mitochondrial dysfunction [39]. In physiological conditions, antioxidant defenses (enzymatic or non-enzymatic) protect brain tissues against oxidative stress cytotoxicity [40,41]. Enzymatic antioxidant defenses include superoxide dismutase (SOD), catalase (CAT), and glutathione peroxidase (GPx). SOD catalyzes the conversion of superoxide radicals into hydrogen peroxide, which is less toxic than superoxide radicals. CAT catalyzes the decomposition of hydrogen peroxide into water and oxygen, thereby preventing the formation of hydroxyl radicals [42]. CAT is particularly abundant in peroxisomes and helps to protect neurons from oxidative damage during stroke. GPx utilizes glutathione as a co-factor to reduce hydrogen peroxide and lipid hydroperoxides into their corresponding alcohols [43]. GPx is crucial for maintaining cellular redox balance and protecting neurons from oxidative stress-induced damage. Non-enzymatic antioxidants include vitamins E (α-tocopherol) and C (ascorbic acid), glutathione (GSH), carotenoids, and flavonoids [44,45]. The Nf2 (nuclear factor erythroid 2-related factor 2) pathway is also considered one of the most critical antioxidant transcription factors in cells [45,46]. Mitochondrial dysfunction plays a significant role in the pathophysiology of ischemic stroke. Oxidative stress disrupts mitochondrial homeostasis, impairing electron transport chain function, causing electron leakage, reducing ATP production, and promoting cytochrome c release, thereby initiating apoptotic cascades and exacerbating neuronal death. So, upon reperfusion, the sudden reintroduction of oxygen amplifies ROS generation by dysfunctional mitochondria, exacerbating tissue injury. 

## 4. Lipids and Ischemic Stroke

### 4.1. Lipids and the Brain

Lipids, as essential components of brain structure and function, play pivotal roles in stroke pathophysiology. The brain is highly enriched in lipids and contains high concentrations of polyunsaturated fatty acids (PUFAs), especially docosahexaenoic acid [47,48]. Docosahexaenoic acid (22:6n-3, DHA), the most abundant PUFA in neuronal membranes, is a bioactive nutrient essential for brain development, learning ability, and memory [49,50]. Lipids play critical roles in maintaining the structural integrity of neuronal membranes, influencing membrane fluidity, permeability, and normal functioning of receptor proteins. PUFAs can be oxygenated into oxylipins, either via a non-enzymatic free radical-catalyzed pathway or an enzymatic pathway, which regulate several biological processes within the brain.

#### 4.1.1. Enzymatic Metabolism of PUFAs

**Figure 1:**
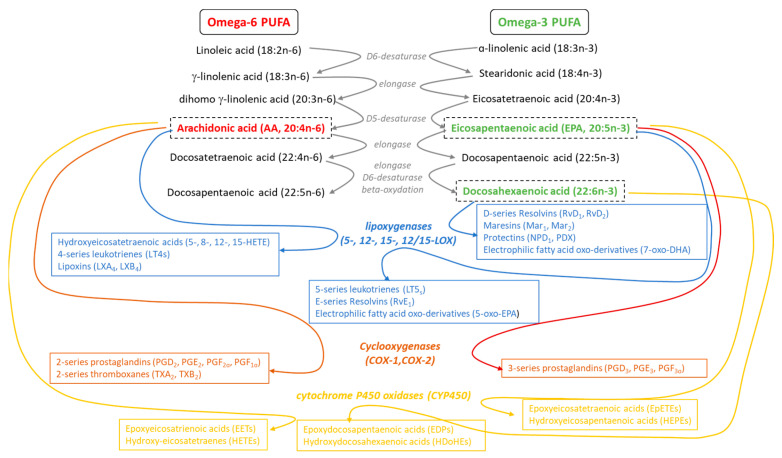
Metabolism of omega-3 and omega-6 PUFAs.

Three main enzymes are involved in the production of oxylipins including cyclooxygenases (COX), lipoxygenases (LOX), and cytochrome P450 monooxygenases (CYP450) [51,52,53] (Figure 1). The biosynthesis of oxilipins is initiated by the initial release of PUFAs from membrane phospholipids, mainly by the action of phospholipase A_2_ (PLA_2_) [54,55]. COX, also named prostaglandin endoperoxide H synthases, possesses both oxygenase and peroxidase activity. COX catalyzes the formation of PGG_2_, a 15-hydroperoxide converted into its 15-hydroxyl derivative PGH_2_ by hydroperoxidase [56,57]. PGH_2_ is then further metabolized to other prostaglandins (D, E, F, I, or prostacyclin), thromboxanes, levuglandins, and hydroxy fatty acids [52,58]. There are two major human isoforms of cyclooxygenase, COX-1, which is constitutively expressed in most tissues, and COX-2, which is induced by inflammatory and proliferative stimuli [59,60]. The main PUFA metabolized by COX is arachidonic acid (AA, 20:4n-6), but COX also oxygenates omega-3 PUFAs like eicopapentaenoic acid (EPA, 20:5n-3). Another prostanoid precursor is dihomo-gamma-linolenic acid (DGLA, 20:3n-6). Lipoxygenase enzymes are a family of dioxygenases, including 5-lipoxygenase (5-LOX), 12-LOX, 15-LOX, and 12/15-LOX isoforms, that catalyze the oxidation of AA, linoleic acid (LA, 18:2n-6), EPA, and DHA, to produce leukotrienes (LTs from AA and EPA), hydroxyperoxyeicosatetraenoic acids (HpETEs) than can be reduced to hydroxyeicosatetraenoic acid (HETEs), lipoxins (from AA), and SPMs (including resolvins Rvs from EPA and DHA; protectins PDs from DHA; and maresins MaRs from DHA) [61,62]. A further class of metabolites generated from omega-3 PUFAs by LOX are the electrophilic fatty acid oxo-derivatives (EFOX), with 7-oxo-DHA, 7-oxo-DPAn-3, and 5-oxo-EPA produced from DHA, DPAn-3, and EPA, respectively [63]. CYP450 oxidases can produce different epoxyeicosatrienoic acids (EETs) and hydroxy-eicosatetraenes (HETEs) from AA, epoxyeicosatetraenoic acids (EpETEs) and hydroxyeicosapentaenoic acids (HEPEs) from EPA, and epoxydocosapentaenoic acids (EDPs) and hydroxydocosahexaenoic acids (HDoHEs) from DHA [64,65].

#### 4.1.2. Peroxidation of Lipids

Lipid peroxidation is a complex process involving the oxidative degradation of PUFAs, leading to the formation of various reactive lipid products and playing a pivotal role in various physiological and pathological conditions. Lipid peroxidation occurs in three consecutive phases [66] as follows: initiation, propagation, and termination. Initiation involves the abstraction of hydrogen atoms from PUFA carbon atoms by ROS or RNS, at the origin of a PUFA radical. Subsequent propagation reactions involve the interaction of lipid radicals with molecular oxygen, forming lipid peroxyl radicals and lipid hydroperoxides. These reactive intermediates can propagate lipid peroxidation through autocatalytic cycles, causing extensive damage to cellular membranes and organelles. The termination of lipid peroxidation occurs through various mechanisms, including radical scavenging by antioxidants, enzymatic degradation of lipid hydroperoxides, and reaction of radical species with each other to give non-radical or non-propagating species.

Isoprostanes (IsoPs) and neuroprostanes (NPs) are prostaglandin (PG)-like compounds that are produced by free radical non-enzymatic peroxidation of AA and DHA, respectively [67,68]. F_2_-isoprostanes and F_4_-NPs are primarily formed in situ from AA esterified in phospholipids and subsequently released [69]. Their formation proceeds via PGH_2_-like bicyclic endoperoxide intermediates, which are reduced to form F-ring IsoPs (F_2_-IsoPs) [67] or undergo rearrangement to form E-ring and D-ring IsoPs [70] and isothromboxanes [71]. Analogous to the formation of IsoPs, the formation of NPs also proceeds through bicyclic endoperoxide intermediates that not only are reduced to F_4_-ring compounds but also undergo rearrangement in vivo to form D_4_- and E_4_-ring NPs [68,72]. Furthermore, a series of highly reactive γ-ketoaldehydes termed isoketals (IsoKs) and neuroketals (NKs) are formed via the isoprostane and neuroprostane pathways, respectively [73,74,75]. IsoKs and NKs have a remarkable proclivity to form covalent adducts in vivo with proteins by binding the ε-amino group of lysine residues, forming lactam and Schiff base adducts, and to crosslink proteins. These γ-ketoaldehydes also covalently modify aminophospholipids, forming pyrrole and Schiff base adducts with phosphatidylethanolamine [76]. All these molecules are detected in the human brain [74]. Malondialdehyde (MDA) and hydroxyalkenals are also aldehydes that are formed during n-3 and n-6 fatty acid peroxidation. 4-hydroxynonenal (4-HNE) is an end-product of the peroxidation of LA, AA, and 15-hydroperoxy arachidonic acid [77]. 4-hydroxy-2E-hexenal (4-HHE) is described as a major degradation product of n-3 PUFA peroxidation, such as DHA [78]. All these reactive aldehydes derived from lipid peroxidation have been suggested to be key mediators of oxidant injury because of their capacity to covalently modify proteins, lipids, and DNA [79,80,81,82,83,84]. 

### 4.2. Dysregulation of Lipid Metabolism in the Pathogenesis of Stroke

Emerging evidence suggests that dysregulation of lipid metabolism plays a pivotal role in stroke pathogenesis and progression. The implication of potent bioactive eicosanoids derived from the arachidonate cascade in ischemic stroke has been attracting more attention.

**Figure 2:**
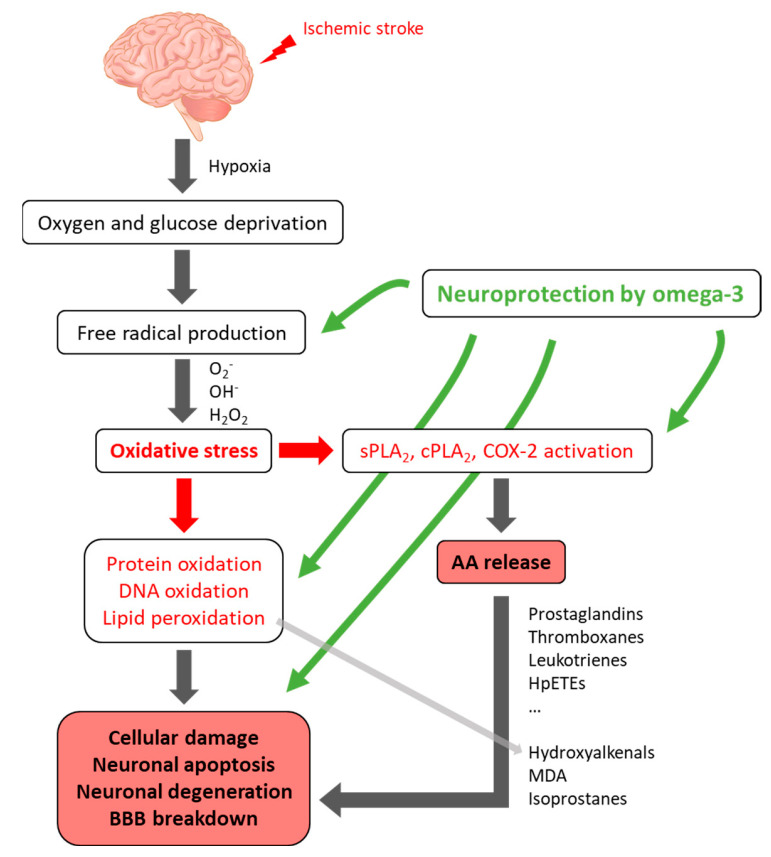
Schematic illustration of the major oxidative mechanisms in ischemic stroke leading to lipid mediators formed by enzymatic or non-enzymatic pathways and playing important roles in various biological processes.

The elevation of intracellular calcium and oxidative stress produced by ischemia/reperfusion in rats activates enzymes including calcium-dependent cytosolic PLA_2_ (cPLA_2_), leading to a rapid release of AA [85] (Figure 2). The release of brain PUFAs from phospholipids by PLA_2_ is exacerbated by the deprivation of oxygen in brain tissue. Plasma PUFAs, including AA, EPA, and DHA, are also increased under ischemic stroke in mice models [86]. Secretory PLA_2_ (sPLA_2_-IIA) is induced in reactive astrocytes in response to transient focal cerebral ischemia by occlusion of the middle cerebral artery (MCA) in the rat brain. sPLA_2_-IIA gene expression is up-regulated in rat brains after transient global ischemia [87]. The infarct size is reduced after the administration of quercitine, a PLA_2_ inhibitor, to MCA-occulted rats [88]. 

COX-2 is also upregulated in the infracted human brain [89] and in vivo models of stroke by occlusion of the MCA [90]. The selective COX-2 inhibitor NS-398 [91,92] reduces the accumulation of PGE_2_ in the post-ischemic brain and ameliorates cerebral ischemic damage [90]. PGD_2_, PGE_2_, PGF_2α_, and TXA_2_ are elevated in stroke [3,6,93]. The effects of PGs on the brain are dependent on their respective receptors. PGE_2_-EP1 receptors contribute to the neurotoxicity mediated by PGE_2_ by increasing the Ca^2+^ dysregulation underlying excitotoxic neuronal death [94]. ONO-AE-248, a selective EP3 agonist, significantly increased infarct size in the MCA occlusion model and aggravated the lesion caused by N-methyl-D-aspartic acid-induced excitotoxicity. Conversely, genetic deletion of EP3 receptor provided protection from acute excitotoxicity [95]. FP receptor of PGF_2α_ has been shown to significantly contribute to excitotoxic brain injury and cerebral ischemia [96].

Lipoxygenase products were also detected in the brain after ischemia [4,97,98]. Concentrations of LTC4 and LTD4, which are potent inflammatory mediators [99], increased in the brain after ischemia [97]. The amounts of 5-, 9-, 11-, and 15-HETEs were significantly increased in ischemic rat brains after MCA occlusion [4]. The amelioration of stroke damage, by decreasing mortality-adjusted infarct size, was observed in FLAP knockout mice that do not produce LTs [100]. 

Markers of oxidative damage are increased immediately after ischemic stroke and remain elevated for several days after stroke onset. IsoPs are increased in different ischemic stroke models. The level of 8-epi-PGF_2_α is more elevated in primary rat cortical neurons exposed to hypoxia followed by reoxygenation as well as in in vivo model (rats submitted MCA occlusion followed by a reperfusion period) [5]. F_2_-IsoP levels are increased in the plasma of acute ischemic stroke patients [101]. 15-A_2t_-IsoP is abundantly produced in infarcted human cortical tissue, and 15-A_2t_-IsoP led to a rapid induction of the mitochondrial permeability transition pore and induced the release of cytochrome c from mitochondria in primary neuronal cultures [102]. 15-A_2t_-IsoP also induces neuronal apoptosis and potentiates oxidative glutamate toxicity in vitro [103]. The levels of F_4_-NPs and HETEs were significantly higher in the plasma of stroke patients [104]. These increases were highest from 6 to 12 h after stroke onset. Elevated levels of urinary 8-iso- and 2,3-dinor-F_2_-IsoPs were observed in stroke subjects, whereas urinary levels of 2,3-dinor-5,6-dihydro-F_2_-isoPs were decreased [104]. 4-HNE concentrations were higher in the plasma of experimental stroke rats with occlusion of the MCA [105]. Moreover, intravenous injection of 4-HNE increased both brain oxidative stress and infarct area induced by ischemia. Guo et al. (2013) [106] showed that mitochondrial aldehyde dehydrogenase 2 (ALDH2), an enzyme detoxifying aldehydes such as 4-HNE, protects against ischemic stroke. Regarding MDA, plasma and serum levels are positively correlated with stroke outcome [107,108,109] and negatively correlated with Mini-Mental State Examination (MMSE) scores [110].

### 4.3. Lipids as Neuroprotective Agents for Ischemic Stroke

#### 4.3.1. Omega 3 PUFAs

The high level of lipids in the brain delineates their critical role in preserving neuronal function and synaptic plasticity. Among them, PUFAs are known to optimize synaptic membrane organization and function, rendering neurons more resistant to neurodegenerative processes. PUFA brain content varies between 25 and 30% and is composed mainly of DHA (12–14% of total fatty acids) and AA (8–10% of total fatty acids) [111,112], both being critical for brain function and contributing to the prevention of age-related neurodegeneration and cognitive deficits [113,114,115]. Omega-3 PUFAs play a key role in stroke neuroprotection. A high EPA/AA ratio was associated with good outcomes in ischemic stroke, suggesting that pre-stroke nutrition habits influence the severity of ischemic stroke in patients [116]. Low DHA level was also shown to be a potential factor affecting the risk of depression in stroke patients [117]. The protective effects of omega-3 PUFAs against damage in stroke patients have been shown to involve multiple pathways. The administration of DHA conferred high-grade neuroprotection in an MCA occlusion model [118,119]. The protective effects of omega-3 PUFAs against damage in stroke patients have been shown to involve multiple pathways. DHA reduces oxidative stress (F_4_-NPs and 8-iso-PGF_2_) following hypoxia–ischemia in the urine of piglets [120]. Post-stroke DHA injection efficiently reduced brain infarct and ameliorated neurological deficits 3 days after transient MCA occlusion, inhibiting the infiltration of immune cells and promoting the polarization of macrophages toward an anti-inflammatory M2 phenotype, therefore reducing central and peripheral inflammation after stroke [121]. DHA can reduce oxidative stress and cerebral ischemia–reperfusion injury by reducing COX-2 expression [122]. In rat brain microvascular endothelial cells under an oxygen- and glucose-deprivation environment (OGD), that mimics ischemic stroke in vitro, DHA was shown to decrease apoptosis, COX-2 protein expression, and the secretion of PGE_2_, PGI_2_, vascular endothelial growth factor (VEGF), and angiopoietin-2 (Ang-2, which has been shown to cause blood–brain barrier damage and to increase endothelial cell apoptosis) [123]. DHA can also decrease hypoxia/reoxygenation injury by activating Src-suppressed C kinase (SSeCKS), a substrate of protein kinase C that plays a key role in maintaining cell morphology and tight junctions of the blood–brain barrier and regulating cell permeability [124,125].

Other PUFAs have been shown to protect against ischemia. EPA inhibits oxidative damage and the inflammatory response after ischemic brain injury [126]. The deuterated form of linoleic PUFA (D4-Lnn) enhances its protective properties against oxidative stress caused by ischemia-like conditions [127]. ALA, the precursor of DHA, was also shown to reduce the consequence of stroke [128]. ALA, delivered either by intravenous injection (iv as a pre-treatment or a post-treatment therapy) or by dietary supplementation, has protective effects against cerebral ischemia [129,130,131,132].

#### 4.3.2. PUFA Derivatives from Enzymatic Pathways

The roles of PUFA mediators in stroke neuroprotection are emerging [133]. Neuroprotectin D1 (NPD1), a DHA metabolite formed via a 15-LOX-initiated mechanism, has been shown to be neuroprotective in the ischemic brain [118,119].

NPD1 inhibits leukocyte infiltration mediated by brain ischemia–reperfusion, decreases proinflammatory gene expression, and increases neurogenesis [118,134]. The combination of resolvin D1 (RvD1) with NPD1 improves neuroprotection after focal cerebral ischemia in rats [135]. The exogenous supply of RvD2 via intraperitoneal injection, another derivative of ω-3 PUFAs, reversed cerebral ischemia–reperfusion injury caused by MCA occlusion by decreasing inflammation, brain edema, and neurological scores [136,137]. The neuroprotective actions of lipoxins (LXs) have been well established. Intracerebroventricular administration of LXA_4_ decreases infarct volume and neurological deficit after MCA occlusion, partly via PPARg agonistic actions and by decreasing inflammation and neutrophil infiltration [138,139,140]. Maresin 1 (MaR1) also protects against ischemia–reperfusion injury by inhibiting pro-inflammatory cytokines and NF-kB p65 activation [141].

#### 4.3.3. Delivery of Omega-3 PUFAs to the Brain

The brain requires a constant supply of DHA from the blood to maintain DHA levels within the brain. Several plasma pools have been proposed to supply the brain with DHA, including plasma lipoproteins, lysophosphatidylcholine (LysoPC), and unesterified fatty acids. LysoPC-DHA is a privileged physiological carrier of DHA across the blood–brain barrier into the brain [142,143,144,145]. Studies showed that dietary DHA as LysoPC, but not as free acid, enriches the brain and improves memory in adult mice [146] and that LysoPC is more efficient than triacylglycerol or phosphatidylcholine in enriching the brain with DHA [147]. A specific protein expressed in brain endothelium called Mfsd2a has been reported to bind LysoPC-DHA, but not free DHA, for its uptake into the brain [148,149,150]. AceDoPC (1-acetyl,2-docosahexaenoyl-glycerophosphocholine), a stabilized form of LysoPC-DHA, targets efficiently and specifically DHA to the brain [151,152,153,154]. It was shown to prevent post-ischemic stroke consequences and to decrease oxidative stress in a stroke animal model of ischemia-reperfusion [155], to decrease the neuroinflammation induced by lipopolysaccharides in mice and microglia cells [156], and to stimulate neurogenesis in an in vitro OGD model of ischemia [157]. AceDoPC decreased the level of AA metabolites involved in oxidative stress and inflammation, including 8-epi-PGF_2_α, PGD_2_, PGF_2_, LTB_4_, and 15-HETE, and activated the nuclear factor erythroid 2-related factor 2 (Nrf2) pathway [157], a critical antioxidant transcription factor in cells upregulating enzymes such as heme oxygenase 1 [158]. DHA carried in triacylglycerol emulsion exhibits neuroprotectin effects in mice subjected to hypoxic–ischemic brain injury [159]. Omega3-rich diets, like fish oil supplementations, combined with intraperitoneal DHA injections, exerted neuroprotective actions on ischemic brain injury [160]. 

The bioavailability and the delivery of omega-3 PUFAs or other drugs to the brain can be improved by nanoparticles. Positive outcomes highlight the efficiency of lipid nanoparticles and nanostructured lipid carriers to target the brain and protect against ischemic stroke [161,162,163]. The intranasal route of PUFAs or other drug delivery via lipid nanoparticles is also very promising, allowing the drug to directly reach the brain by bypassing the blood–brain barrier, a structure that restricts the passage of therapeutic agents and limits the efficacity of the delivery [164,165,166,167].

### 4.4. Lipid Biomarkers in Ischemic Stroke

Lipid-derived biomarkers in stroke research have gained significant attention because of their potential roles as diagnosis, prognosis, and therapeutic targets in stroke management. Different key lipid-derived biomarkers are studied in relation to stroke. This includes lipid peroxidation products (MDA, 4-HNE, IsoPs, and NPs), PLA_2_, and oxylipins, as previously described. Free FAs, as discussed above, also represent a significant predictive factor for the prognosis of ischemic stroke patients. For example, the DHA/EPA ratio has a positive correlation, while the EPA/AA ratio negatively correlates with all prognostic parameters (clinical, paraclinical, and outcome parameters) [168]. Saturated FAs play a negative role in long-term cognitive outcomes in stroke patients [169]. Increased low-density lipoprotein cholesterol (LDL-C) is also considered a relevant marker for ischemic stroke and was significantly associated with an increased risk of ischemic stroke [170,171,172]. However, some studies found no association between high levels of LDL-C and increased risk of ischemic stroke [173]. Non-high-density lipoprotein (HDL) cholesterol (meaning cholesterol of all atherogenic lipoproteins) was shown to be a better marker of risk of ischemic stroke than LDL-C [174]. The ratio of triglycerides (TGs)/HDL-C was significantly higher in young patients with ischemic stroke compared with older cases or healthy adults [175]. The TG/HDL cholesterol ratio was positively correlated and the total cholesterol/TG ratio was negatively correlated with silent brain infarct lesion burden [176], and the total cholesterol to HDL-C ratio was associated with an increased risk of ischemic stroke [169]. Another lipid class considered a biomarker of stroke is LysoPC. Low plasma levels of LysoPC 16:0 are a potential predictor of stroke recurrence [177]. LysoPC was closely associated with stroke recovery [178]. All these studies show an interest in lipidomic profile analyses. Indeed, comprehensive lipidomic analyses have revealed alterations in the overall lipid profiles of stroke patients. These lipidomic signatures may serve as diagnostic and prognostic biomarkers for stroke subtypes and severity. Multi-omics approaches are essential for lipid biomarker research. Opto-lipidomics also opens new opportunities to study lipid biomarkers [179]. Research into lipid-derived biomarkers in stroke is ongoing, and further elucidation of their roles in stroke pathophysiology may lead to the development of novel therapeutic strategies and personalized treatment approaches.

## 5. Conclusions

In conclusion, this review sheds light on the roles of oxidative stress and lipids in ischemic stroke. Lipids play multifaceted roles in stroke pathophysiology, contributing to neuronal injury, oxidative stress, inflammation, and blood–brain barrier dysfunction. Elucidating the specific lipid pathways involved in stroke pathogenesis and evaluating the efficacy of lipid-targeted interventions hold promise for improved outcomes following ischemic events. The identification of reliable lipid-derived biomarkers remains crucial for early diagnosis, prognostication, and therapeutic targeting. Different therapeutic options targeting lipid-derived biomarkers exist such as drugs that target more specifically LDL-C (statins; ezetimide; evinacumab; PCSK9 inhibitors…), the TG/HDL-C ratio (niacin or omega-3 fatty acids...), and oxylipins (acetylsalicylic acid, aspirin, preventing the production of thromboxane by the inhibition of COX and then platelet aggregation; omega-3 fatty acids; selective COX-2 inhibitors…). Edaravone and Dl-3-n-Butylphthalide are two neuroprotective antioxidants that are approved for clinical use. While many promising novel drug therapies are in various stages of preclinical and clinical development, translating these findings into effective treatments for ischemic stroke remains a significant challenge. Rigorous clinical trials are essential to evaluate the safety and efficacy of these therapies in human patients and to bring them to widespread clinical use. Ongoing research is exploring novel therapeutic approaches for ischemic stroke, including stem cell therapy, neurorecovery agents, anti-inflammatory drugs, and neurovascular protective agents. Lipid nanoparticles also hold great promise as drug delivery vehicles in the treatment of stroke, offering targeted delivery, controlled release, and the potential for combination therapy and diagnostic applications. Further research and development in this field are essential to validate the clinical utility of lipid biomarkers, explore their potential as therapeutic targets in ischemic stroke management, and optimize the strategies of administration for stroke treatments.
